# Surgical Club of S.W. England, Meeting at Gloucester and Cheltenham, November 1987

**Published:** 1989-02

**Authors:** 


### Surgical Club of South West England

### Meeting at Gloucester and Cheltenham November 1987

**The afternoon session was combined with a visit of Council of the Royal College of Surgeons of England and followed by the Arris and Gale Lecture on 'Islet Isolation and transplantation techniques in the Primate', by Mr D Gray FRCS (the Radcliffe Infirmary, Oxford).**

### EXPERIENCE WITH RESTORATIVE PROCTOCOLECTOMY

**W H F Thomson**
Gloucestershire Royal Hospital, Gloucester

All twenty-three patients (twenty-two colitics, one polyposis) so managed between 1984 and 1987 were reviewed prior to the co-joined meeting, where the results were presented.

In each patient a 'w' pouch construction was used to optimise volume, with single layer extra mucosal appositional suturing for the same purpose. The rectum was resected in the plane outside its fascia propria (with careful preservation of the autonomic nerves), and removed in toto. Earlier patients underwent complete anal mucosectomy but latterly a one centimetre cuff has been left to preserve the anal cushions.

There were no primary constructive failures, and no mesenteric vessel division was found necessary (to achieve length). There were no leaks, nor was there primary sepsis. Adhesive obstruction occurred and one case of pelvic haematoma requiring operative relief.

The patients were generally delighted. Three functional failures were attributable to poor selection, and two have been reverted to ileostomy; their terminal ileum was, however, successfully reformed from the salvaged pouch. In the remainder, bowel frequency per twenty-four hours was age-related, increasing by one with each decade from an average of three times per day in teenagers. All, however, were continent, defaecated at will, and had lost their urgency.

### CONSERVATIVE MANAGEMENT OF EARLY BREAST CANCER IN GLOUCESTERSHIRE 1980–1986

**J R Owen**
Gloucestershire Royal Hospital, Gloucester

Over recent years improvements in radiotherapy techniques have allowed many woman with so-called early breast cancer to be treated by surgery, conserving the breast, and post-operative radiotherapy. It is exactly fifty years since Geoffrey Keynes pioneered this work. At Cheltenham and Gloucester some three hundred and thirty patients have been treated between 1980 and 1986. T1–30 per cent; T2–53 per cent; T3–17 per cent; NO–79 per cent; N1(b)–21 per cent. The median follow-up time of the group is 36 months; actuarial 5-year survival 77 per cent. There have been thirty three local recurrences in the breast. Twenty one of these had occurred as an isolated local recurrence (6 per cent). Twelve patients (4 per cent), the recurrence occurred after the appearance of metastatic disease. Of the isolated local recurrences, eighteen have been salvaged and three patients have died. 80 per cent of the local recurrences occurred adjacent to the primary tumour site or in a line between the primary tumour site and the nipple. 2 per cent of patients had recurred in the axilla. Cosmesis had been assessed according to a simple four-point scale, excellent, good, fair and poor. 71 per cent of patients fell into excellent or good categories, 22 per cent fair, 7 per cent poor.

**Conclusion**

There is no benefit from more extensive surgical procedures in terms of survival. Local recurrence rates are acceptable at present but a longer period of follow-up is required.

### INTENSIVE CARE ORGANISATION

**R J Eltringham**
Gloucestershire Royal Hospital, Gloucester

The problems associated with the running of a general intensive care unit stem from the wide variety of conditions treated, and to an even greater extent, the large number of consultants treating them.

Intensive Care Staff must establish good lines of communication with relatives, patients and visiting staff particularly when three, four or even more separate teams are dealing with the same patient. The importance of having medical staff readily available on a unit capable of co-ordinating the various teams and ensuring that a coherent picture is presented to the relatives and nursing staff is stressed.

The range of therapy now available means that even in a comparatively modest intensive care unit life can be prolonged almost indefinitely in the face of multi-organ failure. The decision to withdraw support and the timing of this decision raise ethical questions which must be faced although it is often more convenient 'to carry on a little longer'. Relatives do not generally have difficulty accepting the decision to withdraw support providing they are re-assured on two counts: that everything reasonable has been tried but to no avail and that attention will now be directed to making the patients comfortable and allowing them to die with dignity.

Providing intensive care facilities is extremely expensive and utilizes more money than the N.H.S. can provide. The formation of an Intensive Care Unit Charity will not only provide the money for equipment, it will provide an outlet for the surprising number of patients and relatives who feel they wish to make a contribution to this care.

### SETTING UP A C.D.H. SCREENING PROGRAMME

**T P Green, T P B Tasker**
Gloucestershire Royal Hospital, Gloucester

In 1985 the screening procedure for congenital dislocation of the hip was reviewed in the Gloucester Health Authority (annual average birth rate—3,000).

It was found that despite a heavy orthopaedic input, and ninety children a year being treated in a Craig splint, five cases were missed and presented late.

A new protocol was commenced in which more emphasis was placed on a positive family history and other risk factors such as breech presentation or moulded feet, in order to select which children were followed up in addition to those found to be symptomatic at birth.

After the new programme had been running for over a year the results were compared with a similar period under the old system.

Two children have so far been picked up under the new arrangements with a true congenital dislocation, which would have been missed under the old system. Both had been normal at birth but had been selected for follow up because of a positive risk factor.

The new screening programme, although in its infancy, does seem to provide a more logical and thorough screening of children likely to be at risk.

The worries associated with such a programme include the risk of iatrogenically induced hip disorder, prompting the development of non-manipulative screening methods. There is also the possibility of an increasing percentage of births falling into a family history risk group if this is too loosely defined.

### LIMB SALVAGE IN A DISTRICT GENERAL HOSPITAL

**B P Heather**
Gloucestershire Royal Hospital, Gloucester

The vascular workload of a newly appointed surgeon with a major interest in Vascular Surgery was reviewed. 291 new, major vascular cases were seen within the first 20 months of the appointment.

124 patients (43%) presented with critical limb ischaemia, 91 (31%) with claudication and 66 (23%) with aortic aneurysms. Patients with carotid disease represented only 3% of those treated.

Of the 124 patients with critical ischaemia, 78 (63%) were treated by femoro-distal bypass, 22 (18%) or aortic or extra-anatomic bypass, 9 by transluminal angioplasty alone and 15 underwent primary major amputation.

Limb salvage was achieved in 66% of patients having femoro-distal bypass, with an 8% in-hospital mortality. There was no short term difference in success between vein and artificial graft materials.

Aortic or extra-anatomic bypass produced 73% limb salvage but in-hospital mortality was higher in this group at 18%. All post-operative deaths occurred in patients undergoing either aorto-bifemoral or axillo-bifemoral bypass whilst 12 femoro-femoral grafts, most with a simultaneous inflow angioplasty or endarterectomy, were performed with 11 successes and no mortality.

Limb salvage was achieved in 60% of the 124 patients presenting with critical ischaemia. 12 post-operative deaths and 37 amputations (15 as a primary procedure, 22 after failed surgery) accounted for the 40% of failures. A 60% healed below-knee amputation rate was achieved for primary amputations, compared to only 23% when amputation followed failed bypass surgery.

Total vascular operations in the hospital increased from 44 in 1984 to 165 in 1986, while amputations for all 5 surgical teams decreased from 41 to 30 in the same period.

### CULTURAL CONTRIBUTIONS OF COLOPROCTOLOGY

**W H F Thomson**
Gloucestershire Royal Hospital, Gloucester

Surgery's practitioners are the subject of much ribald and pejorative speculation. Of orthopaedics, for instance, it is said you need to be as strong as a horse and at least as intelligent. Flaying more indiscriminately about them other detracters observe that where physicians know everything and do nothing, surgeons know nothing and do everything.

It was, therefore, the speaker's happy task to present the results of a thoughtful archival study which dispelled such unwarranted slurs. Agile and deft in its construction the treatise established coloproctology's fundamental part in the Renaissance in general and in the rectitude of its primacy in the annals of medical history in particular.

Moved by the oratory, convinced by the argument and dazzled by his scope, the audience, responding with generous enthusiasm, endorsed the speaker's contribution to this long-standing and acrimonious debate and the motion was passed nem. con.

The talk ended on a musical note.

### THE USE OF INTRA LUMINAL PROSTHESES IN THE SURGICAL MANAGEMENT OF DISSECTION OF THE THORACIC AORTA

**G Keen**
Bristol Royal Infirmary, Bristol

Although the mortality associated with the surgical management of acute dissection of the aorta has progressively decreased, the overall mortality remains high in the majority of centres, due largely to haemorrhage.

A recent and important innovation has been the introduction of the intra-luminal prosthesis which has particular application in those dissections of the ascending aorta in which the aortic tear is situated well away from the aortic valve and coronary ostia. Its use is particularly attractive in dissection of the descending aorta when the tear is localised. The ease of insertion and low level of bleeding make this a very attractive method of treating these difficult patients.

5 patients are described who underwent insertion of an intra-luminal prosthesis, 4 within hours of acute dissection. 3 had dissections of the ascending aorta and all have survived for at least 5 years and a further 2 patients underwent insertion of the device in the descending aorta with 1 death, this being the patient whose dissection was not acute. Ascending aortic surgery was conducted using full cardiopulmonary by-pass and cold chemical cardioplgia. Descending aortic surgery was conducted using either left atrio femoral bypass or the Gott shunt.

These cases are presented together with a short review of general experience with this device.

### DIAGNOSTIC REAL-TIME ULTRASOUND IN THE HANDS OF THE SURGEON (UROLOGIST)

**S G Vesey, G N Lumb, P J O'Boyle**
Department of Urology, Musgrove Park Hospital, Taunton

Urologist operated real time ultrasound scanning was assessed over the course of 28 out-patient clinics. All ultrasound scanning was performed by a member of the urology team. During the study period a total of 679 patients attended the out-patient clinics and ultrasound scans were performed on 190 (28%) patients. The accuracy of the urologist's scanning was assessed by referring patients for routine x-ray department ultrasound scans. These comparative scans demonstrated that urologist scans were extremely accurate and reliable.

Our experience with out-patient ultrasound has led us to conclude that there are considerable clinical and economic advantages in the routine provision of this type of service. Based on current NHS costs it is estimated that, for the average urology unit, in excess of £100,000 per annum may be saved by the provision of such a service. Similar advantages apply to its use in the general surgical out-patient clinic.

### THORACIC OUTLET COMPRESSION AND THE ROOS OPERATION

**John Fairgrieve**
Cheltenham General Hospital

Thoracic outlet compression is an uncommon condition which may present to the vascular or orthopaedic surgeon, or to the neurologist. The symptoms are due to compression of one or more structures traversing the thoracic outlet i.e. subclavian vein, subclavian artery or the $C_8T_1$ cord of the brachial plexus, and may be due to either anatomical narrowing of the outlet, or to encroachment on it by a cervical rib, fibrous band or anomalous or hypertrophied muscle e.g. subclavius. In this series, 9 cases of cervical rib had been treated by excision, combined with scalenotomy, and all had done well with no significant complications. Cervical rib is a potentialy dangerous condition because of the risk of subclavian aneurysm and embolisation, and excision is usually advisable.

A further 21 patients with T.O.C. were treated by trans-axillary excision of the first rib (Roos operation). Those patients with intermittent subclavian vein compression (3) or neurological compression (3) did well, but 7 out of 15 patients with predominantly intermittent arterial compression or spasm (15) had less satisfactory results. Most of these were young women or teenage girls with poor skeletal musculature. A further 12 patients with acute spontaneous subclavian vein thrombosis (often associated with the oral contraceptive pill and mild trauma) had been treated conservatively with anticoagulants, and all this group did well.

The operative technique of the Roos operation was described in detail, and the indications for exploration of the thoracic outlet discussed.

### SALVAGE AUTOTRANSFUSION IN AORTIC SURGERY

**T Wilson, J Fairgrieve, P Young**
Cheltenham General Hospital

To evaluate our experience with the Solcotrans system of salvage autotransfusion abdominal aortic operations performed between June 1985 and August 1987 were reviewed. During this time 54 patients underwent abdominal aortic surgery. 32 operations were performed for aneurysmal disease (10 following rupture), 21 of the operations were for aortoiliac stenosis and 1 case was to remove an infected aortic graft. 8 cases were excluded because their medical records could not be retrieved or contained inadequate information.

Solcotrans autotransfusion was used in 25 of the remaining 46 patients. Estimated blood loss and blood-bank blood transfusion rates are shown below.

The amount of blood salvaged with the Solcotrans ranged from 400–3250 ml (average 1200 ml representing a mean salvage rate of 42% of estimated blood loss).

The amount of blood-bank blood used was almost halved with the advantages of the salvaged blood being fresh with no risk of incompatibility or disease transmission, immediately available and cheaper per unit than blood-bank blood. However, in emergency situations the Solcotrans proved less effective because of inadequate suction pressures and clotting of pooled blood in non-heparinised patients.

| | *BLOOD LOSS (ml)* | | *BANK BLOOD (ml)* | |
|---|---|---|---|---|
| | *Range* | *Mean* | *Range* | *Mean* |
| No Solcotrans | 700– 6000 | 2850 | 0–5000 | 2600 |
| Solcotrans | 1100–12000 | 2900 | 0–7600 | 1350 |

**The following papers were submitted for the Registrar's Prize, and this was awarded to Mr J M Fleischl.**

### WHICH BALLOON EMBOLECTOMY CATHETER?

**J M Fleischl (W B Campbell)**
Royal Devon and Exeter Hospital, Wonford, Exeter

Seven different balloon embolectomy catheters are now available in Britain, but no data exist comparing their performances. This study tested Size 4 catheters in two different experiments.

On a specially designed mechanical rig catheters of each type were withdrawn nine times through a standard 6 mm lubricated transparent tube containing a fixed stenosis. Balloons were inflated to an initial pressure of 1000±20 cm $H_2O$. Each was then withdrawn at a constant velocity, with continuous measurement of traction force and intra balloon pressure. Marked variation was observed between different balloon types. The range of traction forces was 34–128 g (median 40 g) during withdrawal through the 6 mm tube, increasing to 254–463 g (median 360 g) at the stenosis.

In the second experiment, the volume of fluid and the inflation pressure required to burst balloons of each type were measured, with synchronous video recording of balloon inflation and disruption. Pressures requird to burst different balloon types ranged from 1240–1840 cm $H_2O$ (median 1500 cm $H_20$). Three of the seven balloon types tended to fragment when they burst.

The compliance of balloons may reflect their potential to damage arterial walls, while the tendency of some balloons to fragment when they burst has serious clinical implications. The observations of this study may therefore influence the choice of catheter for embolectomy.

### CONTAINED PELVIC SEPSIS FOLLOWING ANASTOMOTIC LEAKAGE AFTER ANTERIOR RESECTION—WHAT TO DO

**David Deutscher (W H F Thomson)**
Gloucestershire Royal Hospital

Anastomotic leakage following anterior resection, orthograde on-table colonic lavage and anastomotic protection by tube caecostomy often presents late and may, though contained, persist and require complete faecal diversion to allow anastomotic healing and resolution of pelvic sepsis.

At this late stage, faeces has collected above the anastomosis and the formation of a transverse loop colostomy does not prevent the on-going 'seeding' of the pelvic cavity by this faecal column, which, we contend, protracts pelvic sepsis.

We have sought to overcome the problem by clearing out the faeces and the pelvic abscess cavity at the time of creating the colostomy, by a further application of the on-table wash-out technique.

On completion of the colostomy the distal limb of the stoma is intubated with a Foley catheter. The colon above the anastomosis can then be lavaged on the operating table, draining the effluent per anum.

If the anastomotic defect is palpable, the pelvic cavity can also be intubated with a Foley catheter and lavaged until clean.

We illustrate the problem of on-going ill health and pelvic sepsis when the above technique is not used, and contrast the outcome with three reports demonstrating effective curtailment of pelvic sepsis with earlier return to health and hospital discharge when the technique is employed.

*Continued on page 32a.*

### ABSTRACTS OF CLINICAL MEETINGS.
*Continued from previous page.*

**1. Surgical Club of S.W. England**
*Continued from page 26.*

### IDENTIFICATION OF THE SOURCE OF HAEMATURIA BY AUTOMATED MEASUREMENT OF RED CELL VOLUME
**R Banks, S Reynolds, D Hanbury**
Gloucestershire Royal Hospital, United Kingdom

Phase contrast microscopy can identify the source of red cells in patients with haematuria. We have used a standard laboratory Coulter counter to investigate whether mean urinary red cell volume (MCV) would discriminate between glomerular and non-glomerular bleeding. 42 subjects with haematuria were studied in whom a diagnosis had been established by renal biopsy, cystoscopy or radiology. Fresh urine was centrifuged and the sediment resuspended and analysed on a Coulter S+ III. Urinary samples were reanalysed after having remained at room temperature overnight. In the fresh specimens urine erythrocyte MCV ranged from 51 to 148 fl. and changed very little when left overnight. 18 of 21 patients with glomerulonephritis had urinary red cell MCV<80 fl., the lower normal range of blood erythrocytes. 18 of 21 patients with non-glomerular bleeding had red cells in their urine or normal size or larger. Urinary red cells >98 fl. MCV (upper limit of normal for blood erythrocytes) were always from patients with non-glomerular haematuria. Red cells in the urine of patients with glomerulonephritis were always smaller than their own venous blood cells whereas 18 of the 21 patients with non-glomerular lesions had larger urinary than blood erythrocytes. Thus compared with a venous blood sample the finding of smaller urinary erythrocytes predicts glomerulonephritis with a sensitivity of 100% and specificity of 84%. When urine red cell MCV is greater than blood MCV a non-glomerular source is predicted with a sensitivity of 81% and a specificity of 100%. Coulter analysis of urine provides a simple and objective aid to the diagnosis of haematuria.

